# No dynamic changes in the expression of genes related to the epigenetic mechanism during acute exercise

**DOI:** 10.1007/s13353-022-00736-6

**Published:** 2022-11-10

**Authors:** Witold Józef Światowy, Jacek Zieliński, Maria Aleksandra Osielska, Krzysztof Kusy, Dariusz Wieliński, Andrzej Pławski, Paweł Piotr Jagodziński

**Affiliations:** 1 Department of Biochemistry and Molecular Biology, Poznan University of Medical Sciences, Święcickiego 6, 60-781 Poznan, Poland; 2 Department of Athletics Strength and Conditioning, Poznan University of Physical Education, Królowej Jadwigi 27/39, 61-871 Poznan, Poland; 3 Department of Anthropology and Biometry, Poznan University of Physical Education, Krolowej Jadwigi 27/39, 61-871 Poznan, Poland; 4 Department of General, Endocrinological Surgery and Gastroenterological Oncology, Poznan University of Medical Sciences, Przybyszewskiego 49, 60-355 Poznan, Poland; 5 Institute of Human Genetics, Polish Academy of Sciences, Strzeszynska 32, 60-479 Poznan, Poland

**Keywords:** DNA methylation, Epigenetic, Acute exercise, DNMT1, HDAC1, JHDM1D

## Abstract

Physical exercise results in structural remodeling in tissues and modifies cellular metabolism. Changes in gene expression lie at the root of these adaptations. Epigenetic changes are one of the factors responsible for such exercise-related alterations. One-hour acute exercise will change DNMT1, HDAC1, and JHDM1D transcriptions in PBMC. This study examined changes in the expression of genes responsible for epigenetic modifications (HDAC1, DNMT1, and JHDM1D) during and after an incremental exercise test on a treadmill and a 30-min recovery. Blood samples from 9 highly trained triathletes were tested. Examination of the transcripts showed no significant changes. Correlations between transcript results and biochemical indices revealed a significant (*p* = 0.007) relationship between JHDM1D mRNA and the number of monocytes at peak exercise intensity (exhaustion), while there was no significant (*p* = 0.053) correlation at rest. There are no rapid changes in the mRNA levels of the genes studied in blood cells in competitive athletes during acute exercise and recovery. Due to the small group of subjects studied, more extensive research is needed to verify correlations between transcription and biochemical variables.

## Introduction

Gene expression is based on deoxyribonucleotide sequences, and epigenetic mechanisms are responsible for the changes in gene expressions. Several environmental and biological factors can be identified that cause epigenetic modifications. Acute or chronic exercise is among those factors that modify gene transcription and translation. The epigenetic mechanisms are DNA methylation, miRNA expression, and histones alteration, whose common feature is that they alter access to transcription factors. Cytosine methylation impairs access to transcription factors. After pairing with mRNA, miRNA blocks access to transcription factors and can lead to degradation. Histones can be i.a. methylated or acetylated, which leads to greater or lesser availability of DNA for transcription by changing chromatin condensation (Ling and Rönn 2014; Simmonds and Seebacher 2017; Grazioli et al. 2017; Basso and Suzuki (2017)).

The effects of physical activity and training on methylation have already been reported in the scientific publication. Lower levels of global methylation were observed in individuals who declared only 10 min of physical activity per day, compared with those who declared 30 min per day. However, the differences were not significant after taking into account age or BMI (Hughes et al. 2011). Six-month training contributed to greater genome methylation in adipocytes but lower methylation in muscle cells (Barrès et al. 2012) and blood cells with a single round nucleus (PBMC) (Dimauro et al. 2016).

The effects of DNA methylation and histone acetylation on the human body under exercise are better studied than the effects of histone methylation. One example of these less-explored epigenetic mechanisms was a study of protein arginine methyltransferase (PRMT) after acute exercise. The mentioned protein can eliminate the methyl group from the H3K36 region like the enzymes containing the JhmC domain, e.g., (*JmjC domain-containing histone demethylation protein 1 group* (JHDM1D)) (Dimauro et al. 2020).

The changes in *DNA (cytosine-5)-methyltransferase 1* (DNMT1) expression can be of interest. DNMT1 is a maintenance methyltransferase, but its expression decreases with age (Ciccarone et al. 2016). This can be related to the metabolism of IL-6. It was observed that cells stimulated by IL-6 had higher levels of DNMT1 and Il-6 was more strongly expressed after exercise (Horsburgh et al. 2015). Therefore, one can assume that training increases the levels of DNMT1.

IL-6 is a pro-inflammatory cytokine that also has an anti-inflammatory role as it helps to organize the immune system’s anti-inflammatory response during exercise. The general pattern of cytokine release during acute exercise shows an initial increase in IL-6 levels followed by an increase in anti-inflammatory cytokines. IL-6 just induces anti-inflammatory cytokines like IL-1Ra and Il-10. These anti-inflammatory cytokines inhibit pro-inflammatory cytokines. The influence of cytokines on the functioning of the body during exercise and its regeneration after exercise is a very broad topic, and the distinguishing cytokine is IL-6 given its broad role in the body’s adaptation to exercise (Docherty et al. 2022; Moldoveanu et al. 2001; Ostrowski et al. 1999). The epigenetic regulation of the cytokines themselves, which are studied in the literature mainly for cancer, is also important (Yasmin et al. 2015; Zhang et al. 2020; Markopoulos et al. 2019).

Another mechanism suggesting increases in DNMT1 after exercise is the acetylation of the region with DNMT1. Sirtuin 1 (SIRT1) in this gene is responsible for changing the level of acetylation, and oxidative stress reduces the activity of SIRT1 (Dimauro et al. 2020).

Muscle tissue adaptation to exercise also depends on histone deacetylases (HDAC). They affect myocyte enhancer factor-2 (MEF2) or myoblast determination protein 1 (MyoD) (McGee and Hargreaves 2004; Mal et al. 2001). The predominant description in the publications is IIa class HDAC, which is the relocation from the nucleus to the cytosol after exercise. This is how HDAC3 acts after acute exercise, causing greater histone acetylation in the myocyte (McGee et al. 2009). This change does not affect the number of proteins, only their concentration in the nucleus. Similarly, HDAC5 can perform on glucose transporter type 4 (GLUT4) and MEF2 (McGee and Hargreaves 2004). The lack of literature data on the effects of exercise on HDAC1 and the lack of reports showing stable expression in PBMC is the reason to study this molecule.

In this study, we examined molecules related to epigenetic mechanisms with a poorly documented role in adaptation after acute exercise, such as the *DNMT1*, *HDAC1*, and *JHDM1D* genes. We focused on molecules that are expressed in blood cells, such as PBMC, because blood was not sufficiently studied in terms of adaptation to exercise (Nawrocki et al. 2015, 2017).

We tested the hypothesis that incremental acute exercise until exhaustion will enhance DNMT1 (OMIM: 126,375), HDAC1 (OMIM: 601,241), and JHDM1D (OMIM: 619,640) transcriptions in PBMC. What may contribute to epigenetic changes in this tissue is described in the cited publications.

## Methods

The experiment was carried out on nine athletic men aged 23.6 ± 3.6. They have been practicing triathlon for 9.6 ± 1.5 years. With a height of 1.82 ± 0.06 m, they had a relative skeletal muscle index (RSMI) of 8.4 ± 0.8 kg/m^2^. Study participants were presented with the study design and objectives, and written informed consent was obtained. The project was approved by the Ethics Committee at the Poznan University of Medical Sciences (Poznań, Poland) and implemented following the Helsinki Declaration.

### Exercise test

Two days before the participants began the tests, they had to reduce performed physical training. On the day of the laboratory visit, the procedure started with a light breakfast and a 2-h rest. A temperature of 20.5 ± 0.5 °C was maintained in the rooms where the study was conducted. Weight and height were measured using the SECA 285 measuring station (SECA GmbH, Hamburg, Germany). In the main part of the experiment, athletes performed a run with increasing speed until exhaustion on the mechanical treadmill h/p Cosmos Pulsar (Sports & Medical GmbH, Nussdorf-Traunstein, Germany). The test began with 3-min standing on the treadmill and after every 3 min, there was an increase in speed, initially by 4 km/h, then by another 4 km/h, and then increase by 2 km/h at each subsequent stage until interrupted by the participant. After exhaustion, the recovery phase included walking at 4 km/h for 3 min and sitting for 27 min. Cardiorespiratory variables were measured using the ergospirometer (MetMax 3b-R2 ergospirometer). The MetaSoft Studio 5.1.0 software package (Cortex Biophysik GmbH, Leipzig, Germany) was used for data processing. Heart rate was recorded using the Polar Bluetooth Smart H6 monitor (Polar Electro Oy, Kempele, Finland). The devices and software were used following their procedures. At the final stage of the test, maximal oxygen uptake (V̇O_2_max) and the values of accompanying cardiorespiratory variables were determined. V̇O_2_max was considered achieved if at least three of the following criteria were met: (i) a plateau in V̇O_2_ despite an increase in speed and minute ventilation; (ii) blood lactate concentration ≥ 9 mmol/l; (iii) respiratory exchange ratio ≥ 1.10; and (iv) heart rate ≥ 95% of the age-predicted maximum heart rate (Edvardsen et al. 2014).

### Blood sampling

Blood was collected from the antecubital vein using a peripheral venepuncture (BD Venflon Pro, Becton Dickinson, Helsingborg, Sweden). Samples for gene expression determination were taken into monovette with EDTA (S-monovette K3 EDTA, 7.5 ml, Sarstedt, Nümbrecht, Germany), and samples for lactate concentration were collected in lithium heparin monovette (S-monovette, 2.7 ml KE, Sarstedt, Nümbrecht, Germany). Blood was collected after 3 min of standing on the treadmill (rat rest), every 3 min above the speed of 8 km/h, and after 5, 10, 15, 20, and 30 min of recovery. Table 1 shows the blood sampling scheme.

**Table 1:** Time points (stages) at which blood samples were taken

Collection point	Test stage	Total time (min)
1	At rest (before the test)	0
2	Running ‒ at 10 km/h	12
3	‒ at 12 km/h	15
4	‒ at 14 km/h	18
5	‒ at 16 km/h	21
6	‒ at 18 km/h	24
7	Test termination (exhaustion)	24‒27*
8	Recovery ‒ 5 min	32
9	‒ 10 min	37
10	‒ 15 min	42
11	‒ 20 min	52
12	‒ 30 min	62

*The range results from the variation in particpants’ maximum aerobic capacity

### Isolation of PBMC and RNA

PBMCs were isolated on the same day as the treadmill test. For this purpose, the methods with the Ficoll density gradient were used (Lu et al. 2015). The 15-ml tubes were loaded with 1.5 ml Ficoll solution (Sigma), and twice the blood volume was added. The samples prepared this way were centrifuged at 400 g for 35 min at 21 °C. The interphase was collected in a centrifuge tube. Two ml PBS was added and centrifuged at 340 g for 10 min. The resulting pellet was washed two more times with vortex in PBS. The obtained pellet was suspended in Trizol (RiboEx® GeneAll) and stored for further analysis at − 80 °C.

RNA was isolated according to the manufacturer’s procedure for the Trizol reagent (RiboEx® GeneAll) and stored for further analysis at − 80 °C. Qualitative and quantitative controls of RNA preparations were performed using electrophoresis (on a 1% agarose gel) and spectrophotometry making use of the NanoDrop spectrophotometer.

### RT-PCR and qPCR

The isolated substance was prepared for Quantitative polymerase chain reaction (qPCR) by Reverse-transcriptase polymerase chain reaction (RT-PCR). M-MLV Reverse Transcriptase was used for this according to the protocol from Invitrogen. The concentration of total RNA used for RT-PCR reactions was always 100 ng per reaction. From the cDNA obtained after the reaction, 1 µL was collected for the collective standard sample required for the standard curves.

In addition to the selected test genes (HDAC1, DNMT1, JHDM1D), the reactions were also carried out for the ESD and PBGD genes as control and reference to the assessment of the relative quantity. The forward and reverse primer sequences for these genes are presented in Table 2.

**Table 2:** Forward and reverse primer sequences

Gene	Sequence (5′–3′)	Product size (bp)
*DNMT1*	F: GATGAGAAGAAGCACAGAAGT R: TCTTTGGGGGTCGTTTTGCG	149
*HDAC1*	F: GAGACGGGATTGATGACGA R: TGAGCCACACTGTAAGACC	104
*JHDM1D*	F: TCCCTTCACCTACATTTTCTG R: TGCCTGCCTCGCCACATC	89
*ESD*	F: ACCACCAAAGGCAGAAACAG R: GGAGCAATGACAACAAGACC	135
*PBGD*	F: GCCAAGGACCAGGACATC R: TCAGGTACAGTTGCCCATC	160

The prepared cDNA was subjected to a qPCR reaction which was used with the “5X Hot Fire Eva Green qPCR Mix” as described in the product protocol. The reactions were performed on a Lightcycler 480 II from Roche. The correct course of the experiment was confirmed on an agarose gel.

### Statistical analysis

The normality of the distribution was confirmed by the Shapiro–Wilk test. Correlation matrix analysis (Pearson’s correlation coefficients) was conducted to obtain information about the level of significance of the relationships. All statistical tests were performed in the Statistica 13 software package.

## Results

The athletes achieved the following values of basic aerobic capacity indices: V̇O_2_max) = 4.95 ± 0.51 l/min/kg and HRmax = 187.56 ± 6.73 bpm. Blood lactate concentration changed as follows: 1.03 ± 0.20 mmol/l at rest, 10.24 ± 1.67 mmol/l at the moment of test termination (exhaustion), and 4.35 ± 1.72 mmol/l after 30 min of recovery.

**Fig. 1:**
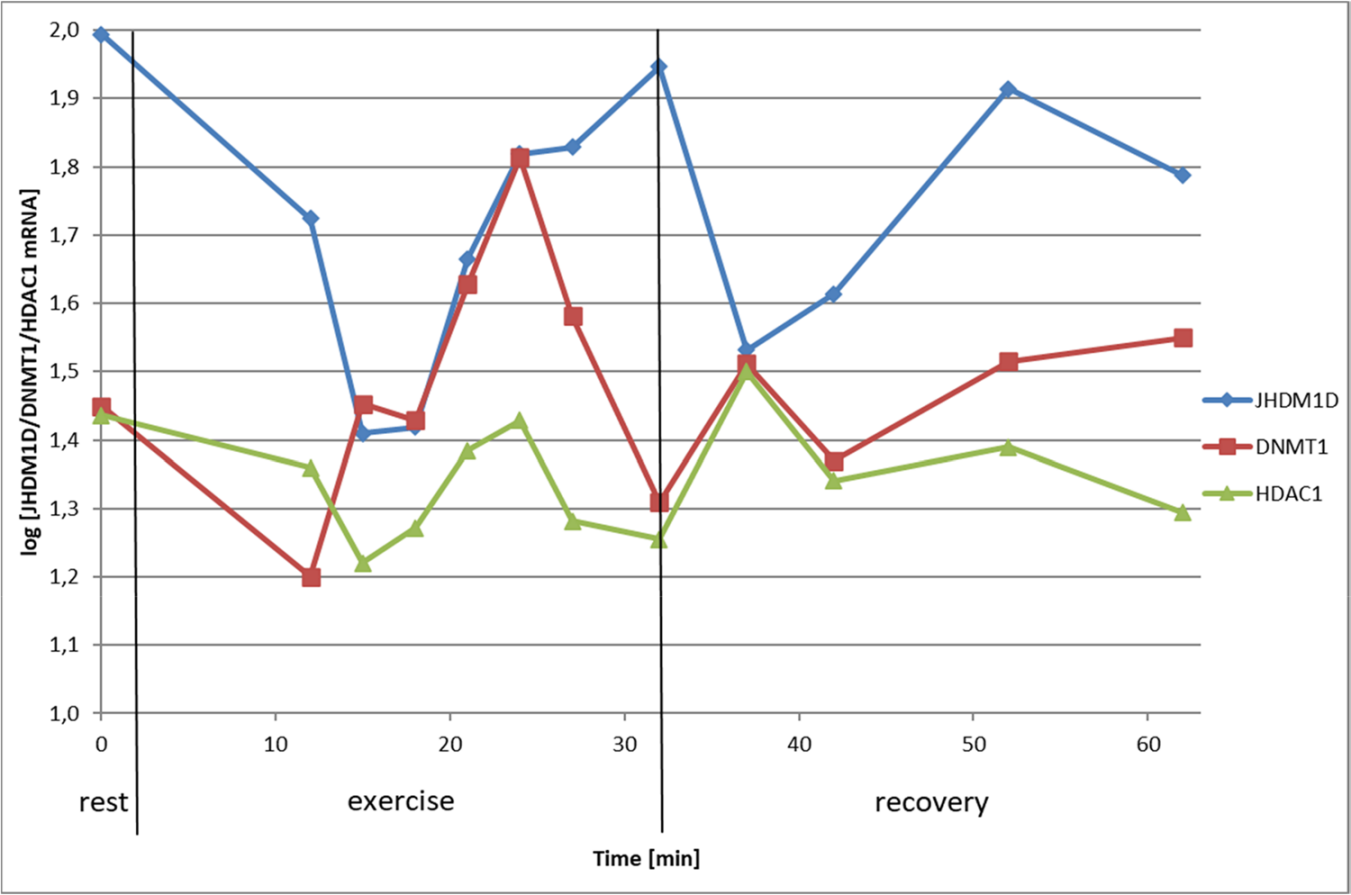
The graph shows the relative mRNA level of the JHDM1D, DNMT1, and HDAC1 genes in PBMC of the tested athletes in relation to the duration of the exercise test and recovery. The value of the transcription rate was determined relative to the reference genes and presented as the logarithm of the multiplicity of the cDNA obtained after the qPCR reaction

During the exercise, the amount of transcripts of the studied genes in blood samples initially decreased and then increased, and these tendencies fluctuated over time, as shown in Fig. 1. Taking into account the high standard deviation and the coefficient of variation in the studied group, presented in Table 3, the changes in the amount of the transcript showed an individual character for each participant.

**Table 3:** Results obtained for individual genes and stages of the exercise test

Phase	Rest	Exercise						Recovery					Mean values
Time (min)/log (gene mRNA)	0	12	15	18	21	24	27	32	37	42	52	62	
Mean HDAC1	1.4361	1.3601	1.2207	1.2716	1.3846	1.4284	1.2816	1.2549	1.4997	1.3408	1.3895	1.2944	**1.347**
Sd HDAC1	0.4882	0.5944	0.4605	0.3610	0.5176	0.4926	0.4762	0.3570	0.4942	0.4501	0.3984	0.4330	**0.460**
v HDAC1 (%)	34.0	43.7	37.7	28.4	37.4	34.5	37.2	28.5	33.0	33.6	28.7	33.5	**34.2**
Mean DNMT1	1.4496	1.2004	1.4531	1.4289	1.6279	1.8138	1.5813	1.3091	1.5119	1.3696	1.5154	1.5495	**1.484**
Sd DNMT1	0.8225	0.6253	0.6721	0.5762	0.8035	0.9224	0.7136	0.5057	0.5817	0.8134	0.7383	0.7886	**0.714**
v DNMT1 (%)	56.7	52.1	46.3	40.3	49.4	50.9	45.1	38.6	38.5	59.4	48.7	50.9	**48.1**
Mean JHDM1D	1.9942	1.7237	1.4095	1.4187	1.6655	1.8187	1.8286	1.9465	1.5314	1.6136	1.9141	1.7869	**1.721**
Sd JHDM1D	1.7629	1.9024	0.7634	0.9711	1.1478	1.4513	1.5970	1.6141	1.0649	1.3600	1.1697	0.9716	**1.315**
v JHDm1D (%)	88.4	110.4	54.2	68.4	68.9	79.8	87.3	82.9	69.5	84.3	61.1	54.4	75.8

Mean levels of gene (JHDM1D, DNMT1, and HDAC1) transcription obtained after the qPCR reactions, the standard deviation (sd), and coefficient of variation (v) are shown

The next step in the statistical analysis was to check the expression outliers in correlation with the corresponding biochemical results. Two of the tested athletes had three times higher mean JHDM1D expression than the remaining subjects. After paying attention to them, it turned out that they differ also in several other biochemical parameters.

An increased level of monocytes distinguished two athletes at time 0 (MonRes)t and after 27 min, i.e., during exhaustion (MonMax), compared to other participants. The number of monocytes and a few other biochemical results were only examined at these two test stages.

Other parameters deviating from the mean in the same athlete were lymphocyte counts (LymRest, LymMax), thrombocytes count (PltRest, PltMax), procalcitonin (PCTRest, PCTMax), and the volume of platelets (MPVRest, MPVMax). Interestingly, while the athletes deviating from the mean in these parameters, one had values above average and the other below average.

Interdependence between molecular and biochemical results in unit times, i.e., at time 0 (MonRes)t and after 27 min of exercise (MonMax), as well as interdependencies of changes that took place over time were calculated.

Statistical significance was observed for the correlation (*r* = 0.82, *p* = 0.007) between the number of monocytes (MonMax) and the amount of JHDM1D transcript after discontinuation of the run (*JHDM1D*Max). There were no significant correlations between monocytes (MonRest) and JHDM1D mRNA (*JHDM1D*Rest) determined at rest (*r* = 0.66, *p* = 0.053) and between changes (MonRest-Max and JHDM1DRest-Max) over time (*r* =  − 0.21, *p* = 0.584).

Another statistically significant correlation (*r* =  − 0,76, *p* = 0.016) was noted for *HDAC1* mRNA and lymphocyte count. The increase in lymphocyte count from rest to the maximum effort was inversely proportional to the decrease in the amount of HDAC1 mRNA detected.

## Discussion

Gene expression and epigenetic changes in the context of exercise are still a topic of research and many studies covering various training programs and exercise trials have been conducted. Most often, they checked changes in these parameters before and after the exercise protocol. The samples are collected before and after the exercise protocol, and the most common biological material is adipose and muscle tissues. These tissues undergo the most significant change during exercise and are the subject of the most common research in the sports context (McGee and Hargreaves 2011; Ling and Rönn 2014; Dimauro et al. 2020). Blood is also often tested due to the less invasive sampling (Horsburgh et al., 2015; Hunter et al., 2019).

One indicator of a tissue activity may be the change in global methylation. Acute exercise has been observed to modulate global methylation by increasing or decreasing it depending on the tissue. This shows that epigenome-altering factors are rearranged, be it by altering the expression of the factors or by modifying the activity (Dimauro et al. 2020).

One of the mechanisms of the body’s adaptation after exercise is epigenetic changes in cells. Exercise reduces the global methylation of the genome in muscle tissue while increasing it in adipose tissue. Acute exercise has been found to increase the activity of the muscular mitochondria due to the hypomethylation of many mitochondrial genes. In terms of regular exercise, a broader importance of hypomethylation of genes related to mitochondria was indicated. The same is also valid for genes related to lipid and glucose metabolism and structural changes, including those responsible for angiogenesis and muscle growth. (Dimauro et al. 2020). There is an epigenetic confirmation that acute and chronic exercise stimulates the muscles to be more active. Adipose tissue is characterized by increased post-exercise methylation, which may indicate that this tissue is less metabolic active. As for blood, its cells lower global methylation after aerobic exercise (Hunter et al. 2019). The results obtained in this study show that acute exercise did not change the amount of mRNA of the studied genes in blood cells.

An increase in DNMT1 transcription was sought because IL-6 contributes to increased protein expression (Hodge et al. 2007, p. 1; Horsburgh et al. 2015). Many studies document increases in blood levels of IL-6 resulting from acute exercise (Fischer 2006). Thus, genome hypomethylation in PBMCs after acute exercise is not related to DNMT1. This confirms the conclusion of Hunter et al. (2019).

Epigenetic modifications of histones promote changes in chromatin condensation. One of the effects of these changes is the increase or decrease in gene expression (Ntanasis-Stathopoulos et al. 2013). Histones, like DNA, can undergo methylation, but in this case, methylation contributes to an increase in hydrophobicity and alkalinity, causing condensation and unavailability of the gene for transcription factors (Rice and Allis 2001). Histone methylation studies in the context of sports activity showed increased H3-K36 lysine methylation in muscles in the absence of movement compared to athletes (Naghavi Moghadam et al. 2019). Interesting observations were made in studies on the influence of acute exercise on Protein Arginine Methyltransferase (PRMT), which is responsible for arginine methylation. It was revealed that acute exercise did not affect the expression of this protein, but it was exported to the nucleus, where the enzyme was more active (Barrès et al. 2012). JmjC domain-containing histone demethylase 1D (JHDM1D) is a methyltransferase whose task is to demethylate lysine. It was described as the target of this demethylation is also histone H3-K36 (Klose et al. 2006). In our research, this enzyme did not change its expression after severe exercise. However, the nuclear transfer mechanism, a common activation mechanism for histone methyltransferases, is possible (McGee and Hargreaves 2004; McGee et al. 2009; Horsburgh et al. 2015).

A well-described muscle protein is Myoblast determination protein 1 (MyoD), which is involved in the growth and reparation of muscle tissue. It was shown that MyoD expression rapidly increased after a single bout of severe exercise (Caldow et al. 2015). This post-exercise effect was also observed after 3 months of resistance training (Mal et al. 2001; Kadi et al. 2004).

HDAC1 is responsible for histone deacetylation and has been shown to reduce MyoD transcription (Mal et al. 2001). With increased MyoD expression in the athletes tested, the amount of HDAC1 transcripts should not increase, which should also be observed in the blood. In other studies, involving obese people, an increase in HDAC transcripts in the blood was observed. However, in the described case of obese people, epigenetic changes related to inflammation may be significant (Dorneles et al. 2016). A similar effect to HDAC1 was observed after acute exercise in research on HDAC2 expression in PBMC (Dorneles et al. 2017). The results obtained in this study showed no change in the amount of HDAC1 mRNA during and after acuter progressive exercise.

A significant increase in leukocytes was observed in our participants during the exercise test. Other researchers described the same effect under similar conditions (Dorneles et al. 2016). The significant relationship between HDAC1 and leukocytes was noticed in these studies, which should be checked in further studies. The relationship between JHDM1D and the number of monocytes also needs confirmation. Further research should be focused on checking the results at the protein level and checking described significant correlations.

The limitation of the study is a small number of participants and samples. Also, the number of transcripts by determining the amount of protein was not verified. The complexity of the translation process and the action of modifying factors such as miRNAs may yield different results. The strong point is the unique approach to collecting samples in multiple units of time, making it possible to verify dynamic biochemical and molecular changes.

## Summary

There was no change in the amount of JHDM1D, DNMT1, and HDAC1 mRNA in the tested PBMCs after acute exercise. The insufficient number of people included in the study may contribute to this. The demonstrated correlation between the amount of JHDM1D transcript and the amount of monocytes, as well as the correlation between lymphocyte count and the amount of HDAC1 transcript should be confirmed on a larger sample.
